# Verrucarin J inhibits ovarian cancer and targets cancer stem cells

**DOI:** 10.18632/oncotarget.21574

**Published:** 2017-10-06

**Authors:** Kelsey Carter, Pranela Rameshwar, Mariusz Z. Ratajczak, Sham S. Kakar

**Affiliations:** ^1^ Department of Physiology, University of Louisville, Louisville, KY, USA; ^2^ Department of Medicine, Hematology/Oncology, Rutgers, New Jersey Medical School, Newark, NJ, USA; ^3^ James Graham Brown Cancer Center, University of Louisville, Louisville, KY, USA; ^4^ Department of Medicine, University of Louisville, Louisville, KY, USA

**Keywords:** Verrucarin J, ovarian cancer, cancer stem cells, securin, tumor recurrence

## Abstract

Ovarian Cancer is the fifth leading cause of death among women from cancer. Cancer stem cells are a small population of cells present in cancer and the cause of chemoresistance and recurrence of cancer. We tested a new compound “Verrucarin J (VJ)”, a metabolite of the *Myrothecium* fungus family, and showed that VJ significantly inhibits cell proliferation of both cisplatin-sensitive (A2780 and OVCAR5) and cisplatin-resistant (A2780/CP70) cell lines in a dose- and time-dependent manner with IC_50_ value of approximately 10 nM after 48 h of treatment. VJ was found to induce apoptosis, DNA damage, and generation of reactive oxygen species (ROS). Treatment of A2780 cells with VJ resulted in a significant suppression of expression of CSCs markers including ALDH1, LGR5, NANOG and OCT4 in a dose-dependent manner, elimination of ALDH1^+^ CSC population and inhibition of expression of Notch1 and Wnt1 signaling pathways. Our study also showed that VJ inhibited the tumorigenic potential (spheroid formation on ultralow attachment plates) of isolated ALDH1^+^ CSCs *in vitro* and tumor growth and metastasis *in vivo*. VJ resulted downregulation of expression of securin an “oncogene” involved in tumor growth and progression, indicating that securin may serve as a downstream signaling gene to mediate antitumor effects of VJ.

## INTRODUCTION

Ovarian cancer is the fifth leading cause of deaths due to cancer among women in the United States [1]. In 2017, over 22,000 women will be diagnosed with ovarian cancer and approximately 14,080 will die from it [1]. The most common treatment for ovarian cancer is cytoreductive surgery followed by chemotherapy consisting of a platinum/taxane combination [2, 3]. Initially, this chemotherapeutic treatment shows a high response rate, however, within 18 to 24 months, most of the patients (70 to 80%) develop resistance to cisplatin resulting in tumor relapse and succumb to their disease within 5 years of their diagnosis [3, 4].

**Figure 1:**
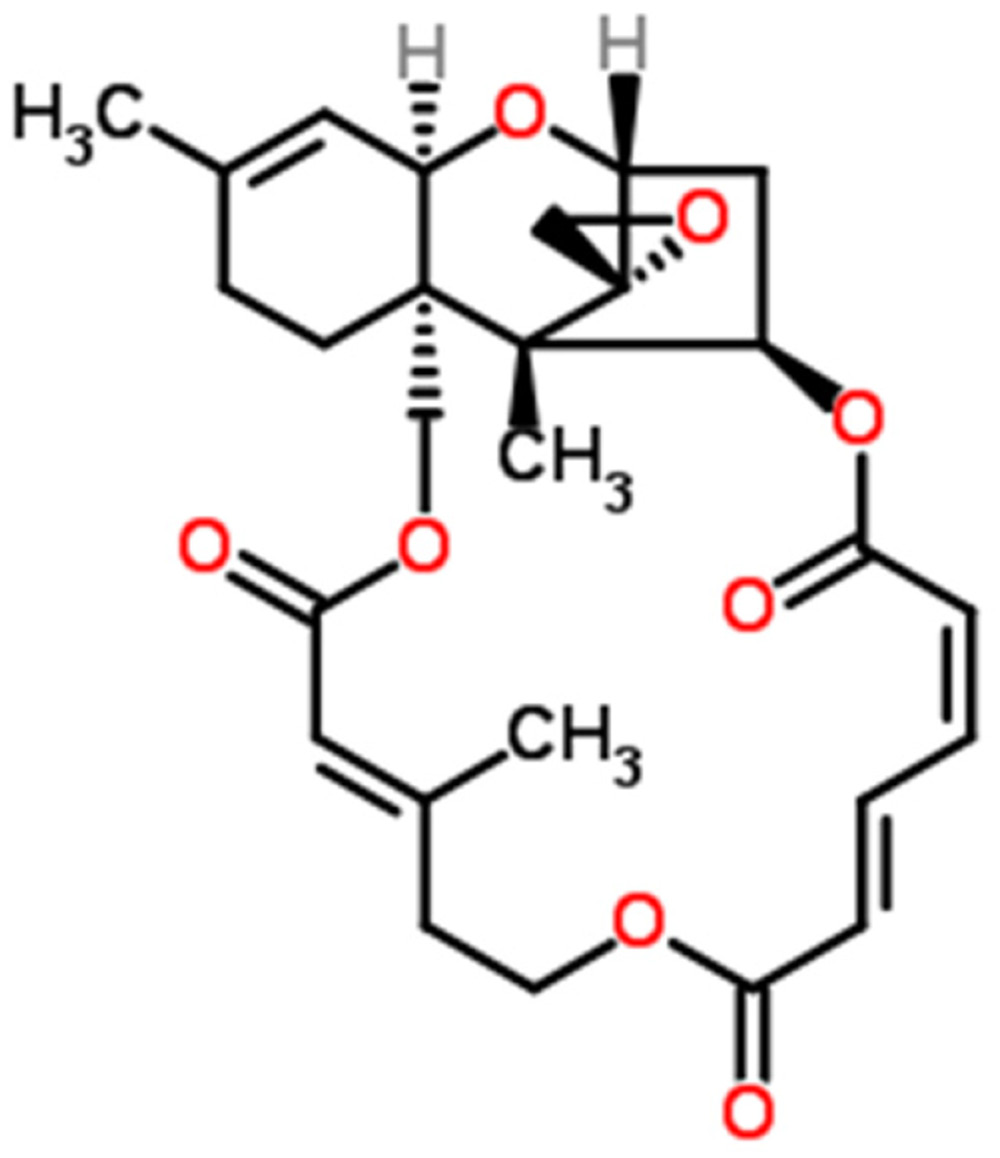
Chemical structure of Verrucarin J

Initial treatments are successful because they target and eliminate cancer cells, however, fail to target cancer stem cells (CSCs). Although CSCs represent only a small population of cells (2-5%) in the tumor, they are reported to be chemoresistant and result in tumor relapse and recurrence [5–9]. Various populations of CSCs have been reported in ovarian tumor cell lines, ovarian tumors, and ascites collected from patients with ovarian cancer [10–18]. In ovarian cancer, the most common CSCs identified include CD24, CD34, CD44, CD117, ALDH1, EpCAM, SSEA4, NANOG, MYD88, and SOX2 positive cells [13]. ALDH1^+^ represents as one of the major populations in ovarian cancer [12]. Increased number of CSCs in ovarian tumors correlate with poor prognosis, including shorter overall life [18, 19]. In recent studies, Abubaker et al. showed that treatment of two ovarian cancer cell lines (epithelial OVCAR433 and mesenchymal HEY) with carboplatin, paclitaxel or combination of both kill the cancer cells but amplify the CSCs [17]. In our previous studies *in vivo*, we showed a significant increase in CSCs: CD24, CD34, CD44, CD117, ALDH1 and OCT4, in tumors collected from animals bearing orthotopic ovarian cancer followed by treatment with cisplatin [20, 21]. This suggests that cisplatin or its derivatives target cancer cells but spares CSCs that undergo amplification leading to chemoresistance and recurrence of cancer. These results clearly indicate that currently used chemotherapies are initially successful, but end up with tumors relapse due to amplification of CSCs. On account of these results, it is obvious that the discovery of a novel therapy that eliminates both cancer cells and CSCs is essential. In our discovery for a novel anti-cancer drug that targets both cancer cells and cancer stem cells, we came across a novel compound “Verrucarin J” (Figure 1) which has not been explored for its anti-tumor properties. As reported below, Verrucarin J (VJ) is a highly potent anticancer drug and targets both cancer cells and CSCs.

## RESULTS

### VJ inhibits cell proliferation

**Figure 2:**
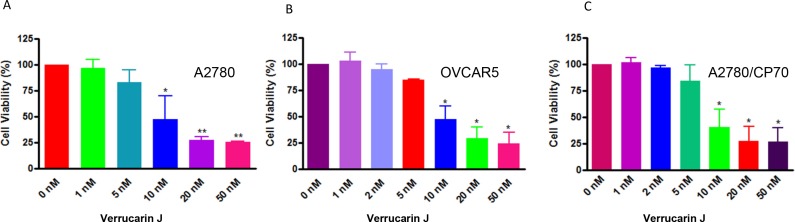
Effect of Verrucarin J on cell proliferation of ovarian cancer cell lines A2780, OVCAR5 and A2780/CP70 Cells were treated with various concentrations of VJ for 48 h. Cell proliferation were assayed using MTT reagent. Results shown are mean ± SD of three independent experiments. ^*^ represents significant at p ≤ 0.05 and ^**^ represents highly significant at p ≤ 0.001.

To determine if VJ has the potential to serve as an anticancer drug we initially performed cell proliferation assays using three different ovarian cancer cell lines: A2780, OVCAR5 (cisplatin-sensitive) and A2780/CP70 (cisplatin-resistance). Cells were plated into 6-well plates and after 24 h, cells were treated with various concentrations of VJ, and cell proliferation was assayed using MTT assays as described previously [22]. Treatment of cells with VJ showed a significant dose-dependent and time dependent inhibition of cell proliferation (Figure 2). IC_50_ values for VJ after treatment of cells for 48 h was found to be approximately 10 nM for each cell line, indicating that VJ is a highly potent anticancer drug and targets cisplatin-sensitive as well as cisplatin-resistant ovarian cancer cells.

### VJ induces apoptosis

**Figure 3:**
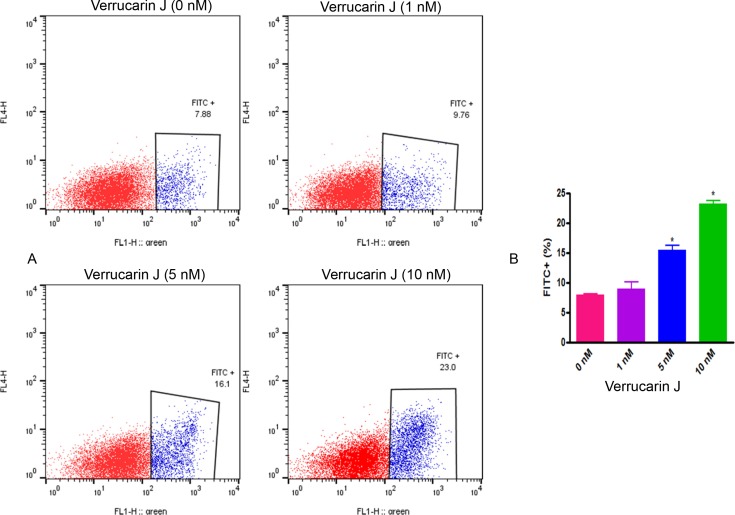
Effect of Verrucarin J on induction of apoptosis A2780 cells were treated with various concentrations of VJ for 48 h. Apoptosis was assayed using Annexin V apoptosis detection kit followed by FACS analysis. **(A)** = FACS analysis. **(B)** = quantitative analysis of apoptosis. The results shown are mean ± SD for the two independent experiments. ^*^ represents significant at p ≤ 0.05. 1X10^6^ cells were used for each analysis. Blue color population of cells represents apoptotic cells.

Apoptosis is a tightly regulated physiological process that is crucial and the most desired solution for cancer therapy [23]. To determine that inhibition of cell proliferation on treatment with VJ is caused by induction of apoptosis, we performed apoptosis assays. We treated the A2780 cell line growing in log phase with various concentrations of VJ for 48 h. After treatment, cells were washed with PBS, stained with Annexin V antibody and analyzed by FACSCalibur. As shown in Figure 3, there was an average of 7.88%, 9.76%, 16.1%, and 23.0% of apoptotic cells when treated with VJ at concentrations of 0, 1, 5, and 10 nM, respectively, suggesting that VJ induces apoptosis in a dose-dependent manner.

### VJ induces DNA damage

**Figure 4:**
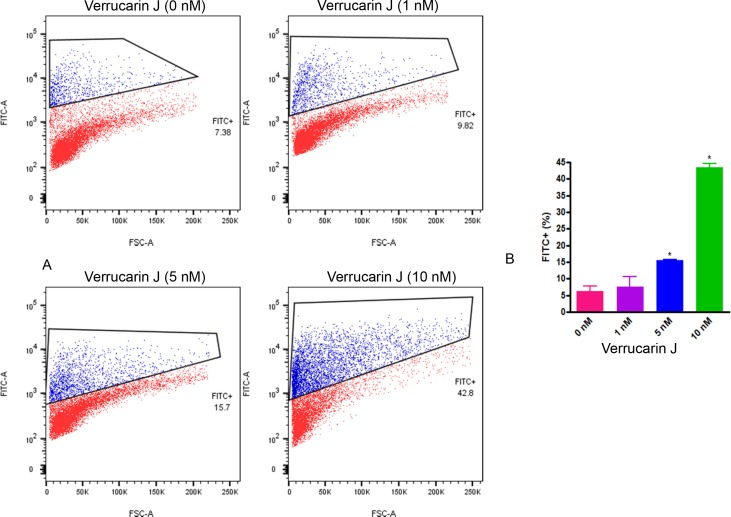
Effect of Verrucarin J on induction of DNA damage A2780 cells were treated with various concentrations of VJ for 24 h. DNA damage was assayed using TUNEL assays using DeadEnd Fluorometric Tunnel Assay System followed by FACS analysis. A = FACS analysis. B = quantitative analysis of cells with damaged DNA. The results shown are mean ± SD for the two independent experiments. ^*^ represents significant at p ≤ 0.05. 1X10^6^ cells were used for each analysis. Blue color population of cells represents DNA damaged cells.

To confirm that VJ induces cell apoptosis through DNA damage, we performed a TUNEL assay. This assay detects the cells that undergo DNA degradation during the late stage of apoptosis, through the use of terminal deoxynucleotidyl transferase [24, 25]. A2780 cells after 24 h of plating as described above were treated with 0 nM, 1 nM, 5 nM, or 10 nM of VJ. After 24 h of plating, DNA damage in response to VJ treatment was assayed using DeadEnd Apoptosis Detection kit (Pharmingen) according to suppliers’ instructions. Treatment of cells with VJ showed a significant dose dependent effect on DNA damage (Figure 4), suggesting that cell death caused by VJ is achieved through DNA damage, hence blocking the cell division.

### VJ generates reactive oxygen species (ROS)

**Figure 5:**
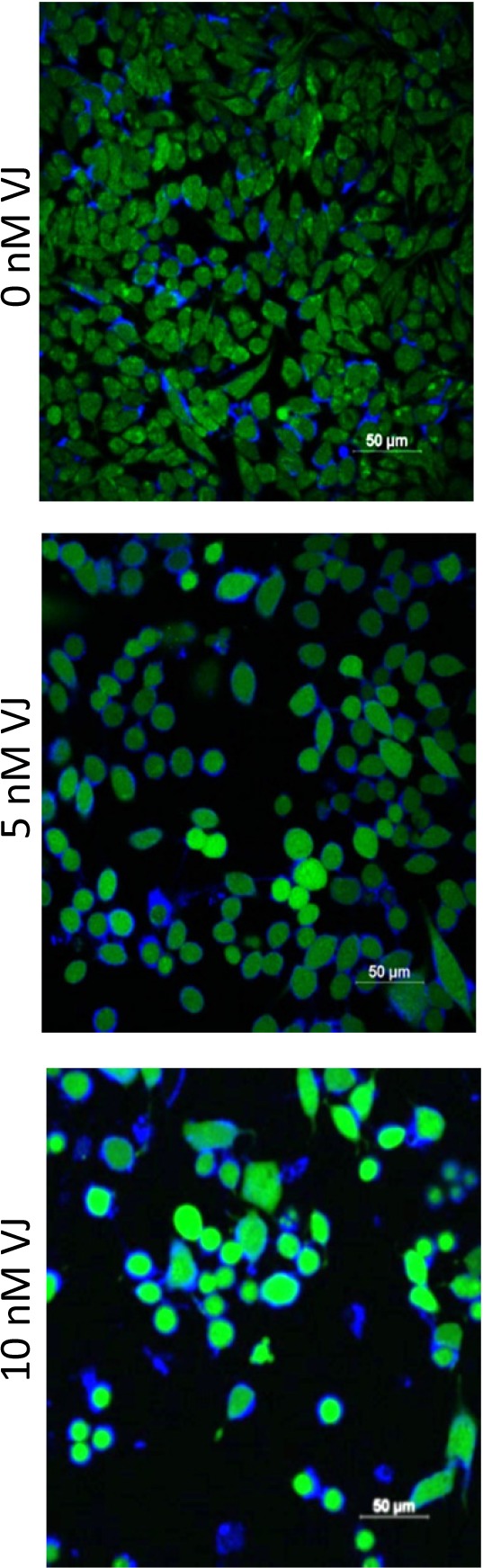
Effect of Verrucarin J on generation of ROS A2780 cells were treated with various concentrations of VJ for 24 h. ROS generation was assayed by incubation of cells with H_2_DCFA for 30 min. Cells were rinsed with PBS and stained with DAPI for nuclei. The cells were examined using confocal microscope and photographed. Green cells show the production of ROS. A significant high level of ROS was observed in cells treated with 5 nM or 10 nM of VJ compared to vehicle treated cells (0 nM VJ). Results shown are representative of three independent experiments.

Generation of ROS is reported to induce apoptosis [26]. To determine if VJ caused apoptosis through generation of ROS, we performed ROS assays using 2′,7′-Dichlorodihydrofluorescein diacetate (H_2_DCFDA). A2780 cells after 24 h of treatment with VJ were treated with H_2_DCFDA (final concentration of 5 μM) for 30 min and then rinsed with PBS and stained for nuclei as described previously [22]. As shown in Figure 5, treatment of cells with VJ caused a dose dependent generation of ROS. Control (vehicle) treated cells showed a few ROS positive cells, whereas, cells treated with 5 nM or 10 nM of VJ showed a large number of ROS positive cells. Cells treated with 10 nM of VJ showed significant cell death, resulting in a loss of cells. The remaining cells appeared to be rounded and apoptotic, and almost 90% of the cells showed high levels of ROS (Figure 5), indicating that generation of ROS by VJ plays a major role in induction of apoptosis leading to cell death.

### VJ suppresses tumorigenic function of ALDH1^+^ CSCs *in vitro*

**Figure 6:**
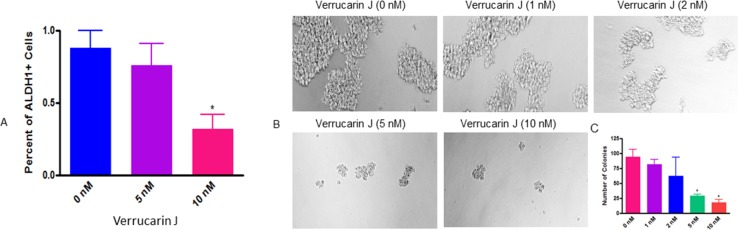
Effect of Verrucarin J on elimination and tumorigenic potential of ALDH1^+^ CSCs **(A)** = A2780 cells were treated with various concentrations of VJ. After 24 h of treatment cells were harvested with trypsin free cell dissociation buffer. ALDH1^+^ cells were analyzed by using Aldefluor kit followed by FACS analysis. The data shown is mean ± SD of three independent experiments. ^*^ represents significance at p ≤ 0.05. **(B)** = ALDH1^+^ cells were isolated from A2780 cell line and plated on ultralow attachment plates. After 24 h of treatment, ALDH1^+^ spheroids were treated with various concentrations of VJ. After 72 h of treatment, spheroids were examined under phase contrast microscope, counted and photographed. **(C)** = quantitative analysis of spheroids on treatment with VJ. The data shown is mean ± SD of three independent experiments. ^*^ represents significance at p ≤ 0.05.

**Figure 7:**
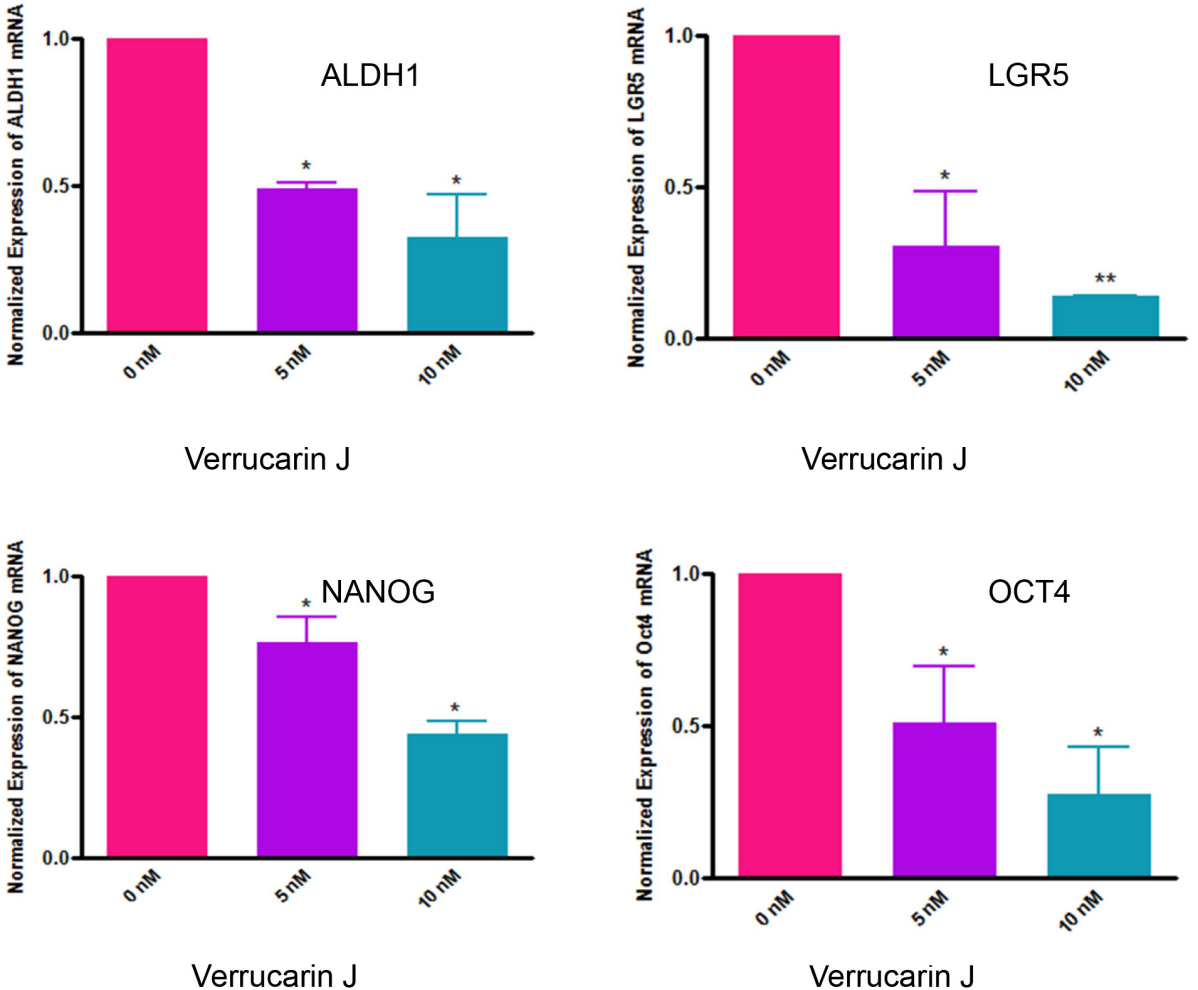
Effect of Verrucarin J on expression of CSCs markers genes expression A2780 cells were treated with various concentrations of VJ. After 48 h of treatment, cells were rinsed with PBS and total RNA was purified. RNA was converted to first strand cDNA, and CSCs markers genes were amplified using the specific primers in real-time PCR. The data shown is mean ± SD for two independent experiments. The data was normalized with GAPDH expression. ^*^ represents significance at p ≤ 0.05, and ^**^ represents highly significant at P ≤0.001.

The most common chemotherapeutic treatment for ovarian cancer is the combination of carboplatin and paclitaxel. Although this treatment kills cancer cells, but increases the CSC population, which leads to reoccurrence of cancer [17, 20]. ALDH1 is one of the commonly studied CSC markers in cancer. It has been demonstrated that high levels of ALDH1 is expressed in tumors, including ovarian tumors, and is the cause of chemo-resistance. ALDH1^+^ cells are highly tumorigenic and enhance stem cells characteristics [27–29]. The expression of ALDH1 is also known to be significantly related to poor clinical outcomes in patients diagnosed with ovarian cancer [23]. To determine if VJ targets ALDH1^+^ CSCs, we treated A2780 cells with various concentrations of VJ (0, 5 or 10 nM). After 24 h of treatment, we assayed ALDH1^+^ CSC population using Aldefluor assay kit, followed by FACS analysis, and expression of ALDH1 mRNA using real-time PCR. FACS analysis of A2780 cells showed a dose dependent decrease in number of ALDH1^+^ cells. Treatment of cells with VJ at 10 nM was found to be significant compared to control vehicle treated cells (Figure 6A). Similarly, real-time PCR analysis of the cells on treatment with VJ showed a dose dependent down-regulation of expression of ALDH1 mRNA (Figure 7). Both concentrations of VJ 5 at nM and 10 nM were found to be significant. These results clearly demonstrate that VJ is highly effective in targeting and eliminating ALDH1^+^ CSC population.

To confirm if VJ directly targets ALDH1^+^ CSC population, we isolated ALDH1^+^ cells from the A2780 ovarian cancer cell line using the Aldefluor kit, as described previously [28]. Approximately 1.0 to 2.0% of cells were found to be ALDH1^+^. The isolated cells were plated on ultralow attachment plates, and within one week of plating, large spheroid (colonies) were formed, confirming the tumorigenic potential of ALDH1^+^ CSCs. To examine the effect of VJ on ALDH1^+^ CSCs tumorigenic potential, the spheroids were collected, dispersed and plated on new 6-well ultralow attachment plates. After 24 h, cells were then treated with various concentrations of VJ. After 72 h of treatment spheroids ≥ 50 mm in size were counted and photographed under a phase contrast microscope. As shown in Figure 6B, VJ caused a dose-dependent inhibition on the formation and size of spheroids. Cells treated with vehicle (DMSO) or lower doses of VJ (1 nM or 2 nM) showed large size and large number of spheroids within 72 h of treatment, whereas higher doses of VJ (5 nM or 10 nM) resulted in formation of very small and a few spheroids compared to untreated (controls). Inhibitory effect of 5 nM or 10 nM on spheroid formation was found to be significant (Figure 6C). The spheroids appeared to be disintegrated and completely apoptotic, suggesting that VJ not only targets cancer cells, but also CSCs.

### VJ suppresses expression of common CSCs markers

As reported above, VJ targets ALDH1^+^ cells. To validate that VJ targets other CSC populations in addition to ALDH1^+^ CSC population, we examined the effect of VJ on expression of genes for NANOG, LGR5 and OCT4, reported to be present in ovarian cancer [12, 13, 30–34]. LGR5 (leucine rich-repeat G protein-coupled receptor 5) has been shown to maintain adult intestinal stem cells, postembryonic development, and is expressed in ovarian cancer [30, 31, 34]. Moreover, studies with colorectal cancer showed a negative effect in the overall patients’ survival if LGR5 is overexpressed [31].

Results as shown in Figure 7 showed a significant and dose-dependent suppression of LGR5 expression in A2780 cells when treated with VJ. NANOG and OCT4 expression was also significantly decreased in a dose-dependent manner. Decrease in expression of mRNAs for ALDH1, LGR5, NANOG and OCT4 suggest reduction in populations of ALDH1, LGR5, NANOG and OCT4 positive cancer stem cells by VJ, suggesting that VJ not only decreases the ALDH1 positive cancer stem cells population but other cancer stem cells population also, translating that VJ not only targets ovarian cancer cells, but also different populations of CSC, suggesting that VJ may reduce/eliminate drug-resistance and hence recurrence of ovarian cancer.

### VJ inhibits CSC self-renewal mechanisms

**Figure 8:**
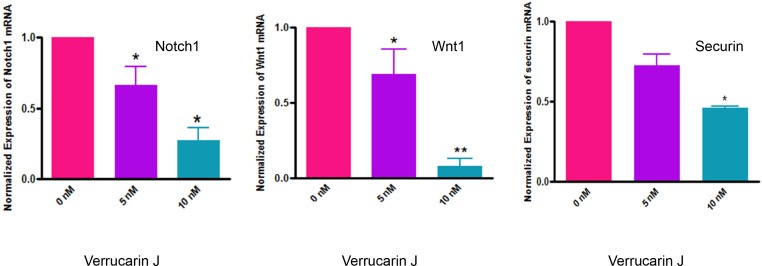
Effect of Verrucarin J on expression of Notch1, Wnt1 and securin genes A2780 cells were treated with various concentrations of VJ. After 48 h of treatment, cells were rinsed with PBS and total RNA was purified. RNA was converted to first strand cDNA. Amplification of Notch1, Wnt1 and securin was performed using specific primers for each gene in real-time PCR. The data shown is mean ± SD for three independent experiments. GAPDH was used as an internal control.

Self-renewal, drug resistance and differentiation are key characteristics of CSCs [35]. Notch1, and Wnt1 signaling transduction pathways have been reported to play major roles in the self-renewal and maintenance of CSCs [35–39]. Notch1 signaling pathway is associated with regulation of cell fate at several distinct developmental stages and has been implicated in cancer initiation and progression [37, 40–45]. In our studies, as shown in Figure 8, treatment of A2780 cells with VJ resulted in a significant down regulation of expression of Notch1 and Wnt1 within 48 h of treatment. This effect was found to be dose dependent (Figure 8), suggesting that VJ blocks the signaling mechanisms involved in self-renewal of CSCs, therefore, may result in reducing or eliminating drug-resistance and hence recurrence of cancer. These results are very interesting and compelling, and strongly suggest that VJ could be a potential therapeutic agent for ovarian cancer.

### VJ suppresses securin expression

To determine the molecular mechanisms by which VJ induces its antitumor effects, we studied the regulation of securin expression in A2780 cell line upon treatment with VJ. Securin, also known as pituitary tumor transforming gene (PTTG), is a multi-domain and multi-functional oncogene that is commonly overexpressed in most tumors analyzed to date. Overexpression of securin results in an increase in cell proliferation, cellular transformation and tumor formation in nude mice [46, 47]. Expression of securin has also been related to tumor progression, invasiveness and malignancy [48]. In our recent studies, we showed co-expression of securin with various CSCs markers (CD24, CD34, CD44, CD117, CD133, ALDH1, SSEA4, LGR5, SHH and β-Catenin) in normal ovary, benign (BN), borderline (BL) and high grade (HG) ovarian tumors (unpublished observations), suggesting an important role of securin in modulating CSC population. Since, securin is a transforming gene, its high levels of expression in CSCs, suggest that it plays an important role in transformation of normal stem cells to CSCs, and down-regulation of securin may result in reduction/elimination of CSCs. In our study, we showed a significant inhibition of securin in A2780 cells on treatment with VJ (Figure 8), suggesting that securin may serve as a downstream signaling gene to mediate VJ anti-tumor function and regulation of cancer stem cell population. However, mechanisms by which securin regulates CSC population remain undecided and are under investigation.

### VJ suppresses tumor growth in nude mice

**Figure 9:**
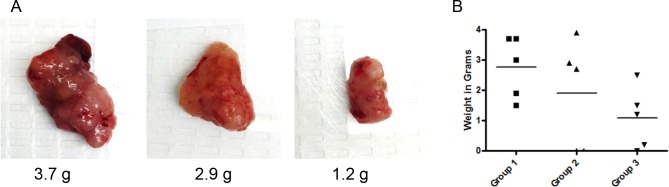
Effect of Verrucarin J on tumor growth in nude animals Tumors were generated in female nude nu/nu (5 to 6 weeks old) mice by injecting A2780 cells (1X10^6^ cells/mouse) i.p. After 10 days of injection of the cells, mice were treated with various concentrations of VJ (0, 0.1, 0.5 or 2.0 mg/kg) for three weeks as detailed in Materials and Methods. After three weeks of treatment, animals were sacrificed, visible tumors in peritoneal cavity and other tissues were collected. Visible peritoneal cavity were weighed. **(A)** = visible tumors (representative of each group). **(B)** = weight of each visible solid tumor in the peritoneal cavity treated with vehicle (control) or after treatment with VJ. Horizontal line represents means of tumors in each group.

Our results, as described above, clearly demonstrate that VJ inhibits ovarian cancer cell proliferation and tumorigenic function of CSC *in vitro*. To determine the antitumor effect of VJ on tumor growth *in vivo*, we generated i.p. ovarian tumors by injecting ovarian cancer cells (A2780) directly into peritoneal cavity. Mice were separated into four groups with five mice in each group. Ten days after injection of cells, mice were treated with a 100 μl of 1) vehicle control, 2) VJ (0.1 mg/kg, 3) VJ (0.5 mg/kg), or 4 VJ (2.0 mg/kg) for three weeks as described in materials and methods. Mice receiving VJ at a dose of 2.0 mg/kg appeared to be sick with swollen belly, body fluid and subsequently died within 3 treatments. We conclude that this dose of VJ (2 mg/kg) as a lethal dose. Mice treated with lower doses (0.1 mg/kg or 0.5 mg/kg) or control (vehicle treated) appeared to be healthy with no loss of activity or appetite throughout the treatment period. In the vehicle treated group, mice formed a large visible tumors in the peritoneal cavity, along with highly visible metastasis to ovaries and intestines (visual analysis). The average weight of visible solid tumors in the peritoneal cavity collected from control vehicle treated animals was 2.9 ± 1.02 g (Figure 9). Mice treated with 0.1 mg/kg showed reduced tumor weight (32% lower compared to control, average tumors weight 1.9 ± 1.79 g), and reduced visible metastasis. Mice treated with 0.5 mg/kg showed a significant reduction in visible peritoneal tumors (61% lower compared to control group) and highly reduced visible metastasis (average weight of tumors 1,08 ± 1.01 g). Visual examination of metastasis to ovaries, we observed presence of enlarged tumors in the ovaries with an average weight of 0.92 g in animals treated with vehicle, whereas, animals treated with VJ (0.5 mg/kg) showed reduced ovarian tumor weight by 71%, suggesting that Verrucarin J is a highly potent anticancer drug and suppresses tumor growth and metastasis. Detail histophatholical analysis of the ovaries and intestines is in process to define the effect of VJ on tumor metastasis.

## DISCUSSION

Currently used therapies for ovarian cancer are initially successful, however, most of the patients (70 to 80%) develop chemo-resistance and recurrence of cancer, which can no longer be eliminated by the previous treatments, and as a result patients succumb to their disease [3, 4]. The reason for chemo-resistance and recurrence of cancer has been linked to presence of CSCs in tumors. Even though CSCs make up a small percentage (2 to 5%) of the tumor cells, current treatments target cancer cells but not the CSCs which undergo amplification and result in recurrence of cancer with characteristics of the original cancer. Therefore, there is an urgent need for a novel therapy that target both cancer cells and CSCs.

In our previous studies we explored the possibility of using combination therapy by combining a natural compound “Withaferin A” with “cisplatin”, a commonly used drug for most of the cancers including ovarian cancer [20] or “DOXIL” (second line chemotherapy) [28]. Although these combinations target both cancer cells and CSCs. In our search for a compound that could be highly potent and could be used in lower dose to avoid unwanted side effects, and potential toxicity, we came across “Verrucarin J” which is a metabolite from the Myrothecium fungus family. To the best of our knowledge, the antitumor effects of VJ, as well its application to target cancer stem cells, have not been demonstrated. In our studies, we showed for the first time, that VJ inhibits cell proliferation of both cisplatin-sensitive and cisplatin-resistance cell lines in a dose- and time-dependent manner with IC_50_ values of approximately 10 nM (Figure 2), suggesting that VJ is a highly potent anticancer drug. Consistent with other commonly used anticancer drugs, VJ also achieves its antitumor effects through induction of apoptosis, generation of ROS and induction of DNA damage (Figures 3–5).

In almost all cases, recurrence of cancer is a major clinical problem which ultimately leads to patients’ death. Approximately, 30% of patients develop cisplatin resistance after first round of chemotherapy. In recent years, presence of small population of CSCs in solid tumors including ovarian cancer has been reported to be responsible for drug resistance and recurrence of cancer [5–9]. Therefore, discovery of a chemo-therapeutic agent that targets both cancer cells and CSCs is mandatory. In our studies, as shown in Figure 2, VJ was found to be highly potent in inhibiting both cisplatin-sensitive and resistant cells line, suggesting that patients that develop cisplatin-resistance as a result of first round of chemotherapy (cisplatin or combination of carboplatin and paclitaxel) may be benefited by VJ. In our studies, as shown in Figures 6 and 7, we showed that treatment of A2780 cells with VJ results in a dose dependent suppression of expression of various CSC markers (ALDH1, LGR5, NANOG and OCT4) and elimination of ALHD1^+^ CSCs. In our studies, we also showed that VJ inhibits the tumorigenic potential of isolated ALDH1^+^ CSC *in vitro* (Figure 6) and tumor growth and metastasis of xenographed ovarian tumors in nude mice generated by injecting A2780 cells (Figure 9), suggesting that VJ not only targets cancer cells but also CSC populations.

Self-renewal, drug resistance and differentiation are key characteristics of CSCs. Sonic Hedgehog (Shh), Notch1, Wnt1 signaling transduction pathways play major roles in the self-renewal of CSCs [36–40, 49]. Notch1 signaling pathway is associated with regulation of cell fate at several distinct developmental stages and has been implicated in cancer initiation and progression [36, 41–45]. In our studies, as shown in Figure 8, treatment of A2780 cells with VJ resulted in a significant down regulation of expression of Notch1 and Wnt1 within 48 h of treatment which was found to be dose dependent, suggesting that VJ blocks the signaling mechanisms involved in self-renewal of CSCs, therefore, may result in reducing or eliminating drug-resistance and hence recurrence of cancer.

The downstream signaling pathways by which VJ induces its antitumor effects remains unknown. In our attempts to define the mechanism, we explored the involvement of securin in regulating the antitumor function of VJ. Securin is an oncogene which is highly overexpressed in most of the tumors including ovarian tumor analyzed to date. The silent observations from our study are that treatment of ovarian cancer cell line A2780 with VJ resulted in downregulation of expression of securin as well as CSCs markers in a dose dependent manner (Figures 7 and 8). In another independent study, we observed that securin is co-localized with CSCs markers such as CD24, CD34, CD133, ALDH1, OCT4, SSEA4, SHH, β-Catenin and LGR5 (unpublished observations) in the normal ovary, BN, BL and HG ovarian tumors, suggesting that there exists a relationship between securin and CSC population. Securin is a transforming gene, therefore it is possible that it may transform normal stem cells to CSCs. Consistent with the effect of VJ in down regulation of CSCs markers and securin, we hypothesize that securin may serve as a key downstream signaling gene to induce its effects through the regulation of CSC population.

## MATERIALS AND METHODS

### Ethical statement

Animals work reported in this manuscript was performed after the approval of the protocol by the University of Louisville Animal Care and Use Committee (IACUC). It is to confirm that all experiments were performed in accordance with relevant guidelines and regulations.

Human epithelial ovarian tumor cisplatin-sensitive (A2780) cell line was obtained as a gift from Dr. Denise Connolly (Fox Chase Cancer Center, Philadelphia, PA). Cisplatin-resistant (A2780/CP70) cell line was derived from A2780 cell line after treatment with cisplatin and obtained as a gift from Dr. Christopher States (University of Louisville, Louisville, KY). OVCAR5 is a human epithelial carcinoma cell line of the ovary, which is derived from the ascites fluid of a patient with progressive ovarian adenocarcinoma without prior cytotoxic treatment was purchased from American Type Culture Collection (ATCC). All three cell lines were cultured in RPMI medium (Sigma) containing 10% fetal bovine serum (FBS) (HyClone), 1% Penicillin/Streptomycin (Sigma), and 0.05% (v/v) insulin (Sigma). Verrucarin J was purchased from AnalytiCon Discovery and DMSO was purchased from Sigma. Verrucarin J was prepared in DMSO.

### Cell proliferation

Cell lines A2780, A2780/CP70, and OVCAR5 growing in log phase were rinsed with phosphate-buffered saline (PBS) (Sigma), trypsinized, and seeded into 96-well plates (3,000 cells/well in a final volume of 100 μl). After 24 h of plating, medium was replaced with fresh medium containing 5% FBS, and cells were treated in triplicates with various concentrations of VJ (0, 1, 5, 10, 20 or 50 nM). Following 24, 48 and 72 h of treatment, medium was replaced with fresh 100 μl of medium containing 20 μl of MTT reagent (CellTiter96 System, Promega) and incubated at 37°C for approximately 30 min to 1 h. Cell proliferation was assessed through color development using ELISA reader at 492 nm as described previously [21].

### Cell apoptosis assays using flow cytometry for Annexin V

A2780 cells were plated into T-75 flaks as described above. After 24 h of incubation at 37°C, cells were treated with VJ (0, 5 or 10 nM), for 24 h and were harvested. Cells were collected by centrifuging at 1,500 rpm for 5 min and resuspended in Annexin V binding buffer (FITC Annexin V Apoptosis Detection Kit, BD Pharmingen) according to supplier's instructions. To insure that cells were not clumped and were single cells, they were passed through nylon mesh and diluted to a final concentration of 10X10^6^ cells/ml. The 100 μl of cell suspension (1X10^6^ cells) was used for each assay. For each concentration of VJ, we used an independent control. To each sample except controls, 2 μl of Annexin V from the kit was added, and was incubated for 15 min at room temperature in the dark. After completion of incubation, 400 μL of binding buffer was added and immediately analyzed by FACSCalibur (BD BioSciences). The stained (and unstained) cells were analyzed using FlowJo software.

### DNA damage using tunnel assay

A2780 cells were plated into T-75 flasks. After 24 h of plating, cells were treated with VJ (0, 5, or 10 nM) and incubated for an additional 24 h as described above. Medium was aspirated and cells were rinsed with PBS, and harvested using trypsin. Cells were centrifuged at 1,500 rpm for 5 min, and then resuspended in 500 μl of PBS, and assayed for DNA damage using the DeadEnd Fluorometric TUNEL System from Promega according to manufacturer's instructions. 1X10^6^ cells were analyzed using BD FACSCalibur and analyzed using FlowJo software.

### Reactive oxygen species (ROS) generation

A2780 cells were seeded into 35 mm glass bottom dishes (FluoroDish, World Precision Instruments) and incubated at 37°C for 24 h, and treated with various concentrations of VJ (0, 5 or 10 nM) as described above and incubated at 37°C. After 24 h of treatment, medium was replaced by fresh medium containing H_2_DCFDA at a final concentration of 5 μM. Cells were incubated at 37°C for 45 to 60 min and then gently rinsed with PBS. Fresh 1.0 ml PBS containing DAPI for nuclei staining was added to the dishes and incubated for 10 min at 37^°^C. Cells were rinsed with PBS and examined under Nikon confocal microscope and photographed.

### Spheroid formation

ALDH1^+^ CSCs were isolated from ovarian cancer cell line, A2780 as described previously [28]. ALDH1^+^ cells were plated into ultralow attachment plates. After 5 to 7 days when large spheroids were formed, spheroids were collected and dispersed mechanically to single cells. The cells were plated into 6-well ultralow attachment plates. After 24 h of plating, small size spheroids were formed and treated with various concentrations of VJ (0, 1, 2, 5 or 10 nM). After 72 h of treatment, spheroids were examined under Olympus inverted phase contrast microscope. The spheroid ≥ 50 mm in size were counted and photographed as described previously [28]. Several images were taken at random field for each treatment.

### Determination of ALDH1^+^ population

A2780 cells were plated into T-75 flasks and incubated for 24 h. Medium was replaced with fresh medium, and cells were treated with various concentrations of VJ (0, 5, or 10 nM) and incubated for 24 h. Cells were washed with PBS and 5 ml of cell dissociation buffer was added to each flask and incubated for approximately 45 min. After incubation, cells were collected by centrifugation at 1,500 rpm for 3 min, then resuspended in 500 μl of binding buffer (Aldefluor, Stem Cell Technologies kit) at 2 X 10^6^ cells/ml and treated with Aldefluor substrate. The negative controls were incubated with DEAB in addition to Aldefluor substrate as described previously [28]. Percentage of ALDH1^+^ cells in each sample were calculated using the MoFlo cell sorter.

### Expression of genes using real-time PCR

To examine the expression of CSCs markers genes, we determine the expression of each marker gene using real-time PCR and specific primers for each gene (Table 1). A2780 cells were plated into T-75 flasks. After 24 h of plating, medium was replaced by fresh medium containing 5% serum and treated with various concentrations of VJ (0, 5, or 10 nM). After 48 h of treatment, cells were harvested by scrapping off in PBS and total RNA from each sample was purified and quantitated as described previously (28). The first strand cDNA was prepared using the iScript cDNA synthesis kit from Bio-Rad. The first cDNA prepared from each sample was subjected to gene amplification in real-time PCR using the standard protocol. C_t_ values were normalized with C_t_ values for GAPDH used as an internal control. The specific primers used for each gene are listed in Table 1.

**Table 1:** Primers sequences for various genes in real-time PCR

Gene	Forward	Reverse
ALDH1	GCACGCCAGACTTACCTGTC	CCACTCACTGAATCATGCCA
LGR5	GCAAACCTACGTCTGGACAA	TGATGCTGGAGCTGGTAAAG
NANOG	ACTCTCCAACATCCTGAA	TTCTGCCACCTCTTAGAT
OCT4	CGCTGGCTTATAGAAGGT	ACAGGTGTCATAAGAATGGATA
NOTCH1	TCAGCGGGATCCACTGTGAG	ACACAGGCAGGTGAACGAGTTG
WNT1	CTCTCTTCTTCCCCTTTGTC	AACTCGTGGCTCTGTATCC
SECURIN	TCGAATTCGACCTGCAATAATCCAGAAT	GCTTTAACAGTCTTCTCAGT
GAPDH	TGATGACATCAAGAAGGTGGT	TCCTTGGAGGCCATGTGGGCC

### Generation of tumors in nude mice and treatment with Verrucarin J

To determine the effect of VJ on tumor growth *in vivo*, we generated intraperitoneal (i.p.) tumors in nu/nu 5 to 6 week old mice (NCI). A2780 cells growing in log phase were harvested, centrifuged and then resuspended in RPMI medium with no serum and antibiotics (10X10^6^ cells/ml). Cells 1X10^6^ in a 100 μl volume were injected into peritoneal cavity as described by us previously [28]. After 10 days of injection of cells, animals were divided into 4 groups (5 animals/group). Group 1 was injected (i.p.) with vehicle (10% DMSO, 90% glyceryl trioctanoate), groups 2, 3 and 4 were injected i.p. with VJ dissolved in vehicle (0.1 mg/kg, 0.5 mg/kg or 2.0 mg/kg). Control as well as experimental groups were administered at every third day for three weeks. At the end of the study, animals were weighed and sacrificed. Visible peritoneal solid tumors, ovaries, kidneys, liver, lungs, and intestine were collected. Visible peritoneal solid tumors and ovaries were photographed and weighed.

### Statistical analysis

Student's t-test was performed to calculate the statistical differences between the control and the treated groups. P ≤ 0.05 was considered statistically significant while p ≤ 0.001 was considered highly significant. The error bars represent the standard deviation of two to three independent experiments.

## CONCLUSIONS

**Figure 10:**
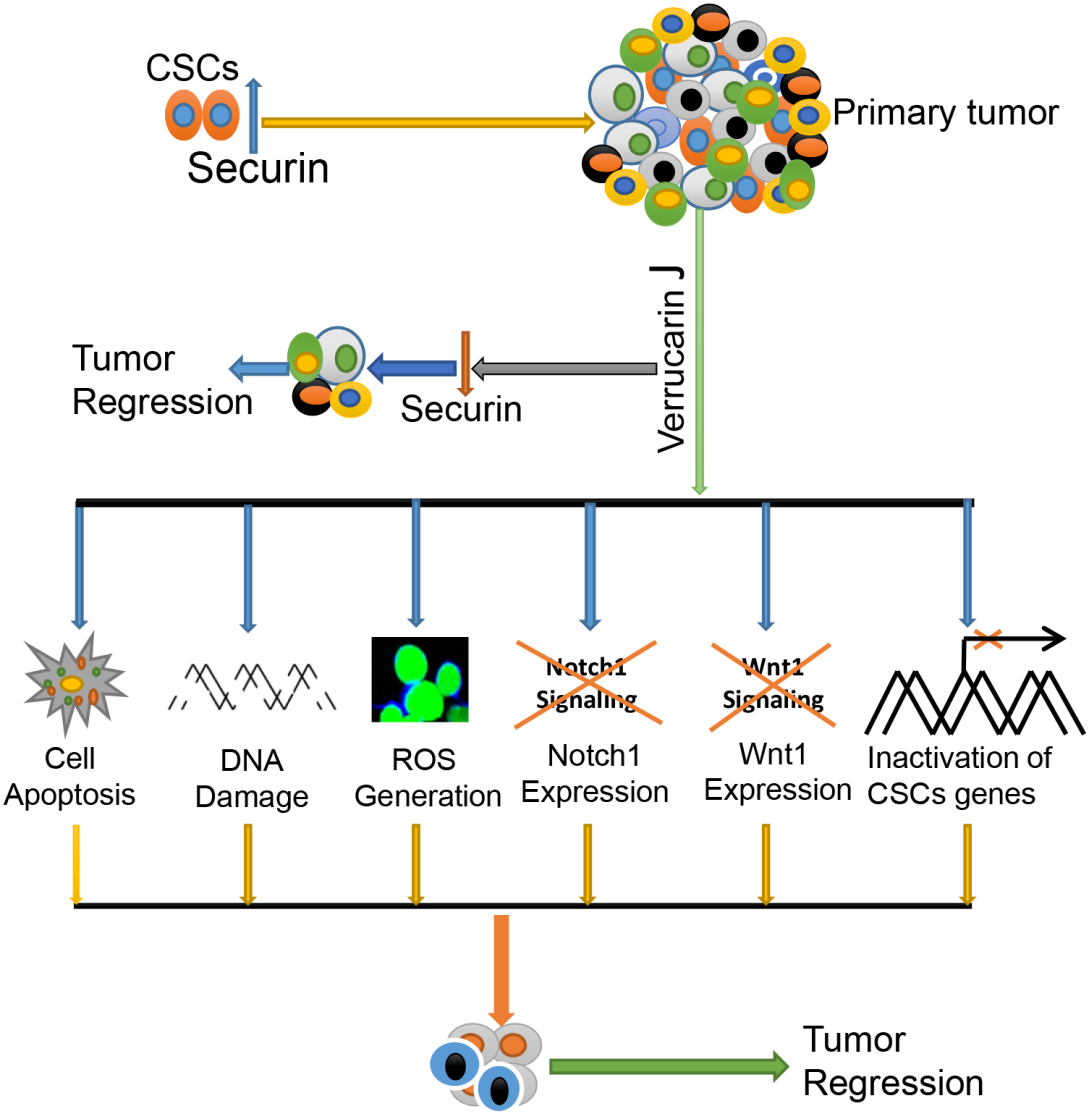
Hypothetical pathway demonstrating function of Verrucarin J on targeting of cancer cells and CSCs

Our studies reveal the potential application of Verrucarin J as a novel therapeutic that targets both cancer cells and CSCs through the regulation of self-renewal signaling pathways (Notch1 and Wnt1), and oncogene “securin”, and hence reducing/eliminating the CSC population leading to reduction of drug resistance and recurrence of cancer (Figure 10).
